# Cricopharyngeal myotomy for delayed Cricopharyngeal dysfunction after head and neck surgery – case report

**DOI:** 10.1186/s12893-019-0667-5

**Published:** 2020-01-08

**Authors:** An Sung, Ka-Wo Lee

**Affiliations:** 1 Department of Otolaryngology-Head and Neck Surgery, Pingtung Hospital of Ministry of Health and Welfare, Zi-You Road #270, Pingtung City, 90054 Taiwan; 2 Department of Otolaryngology-Head and Neck Surgery, Kaohsiung Medical University Hospital, Kaohsiung Medical University, Kaohsiung City, Taiwan

**Keywords:** Cricopharyngeal dysfunction, Cricopharyngeus muscle, Cricopharyngeal myotomy, Vocal cord paralysis, Thyroplasty

## Abstract

**Background:**

Head and neck surgeries can perturb normal structures of neck muscles and nerve innervations, which are supposed to function in harmony to allow complicated process like swallowing. It is still likely that cricopharyngal dysfunction emerges years after the head and neck surgeries.

**Case presentation:**

We report a case with history of left unilateral vocal cord immobility and development of dysphagia and aspiration 2 years after radical thyroidectomy with neck lymph nodes dissection and medialization thyroplasty. Cricopharyngeal dysfunction was impressed and was confirmed with visualization of cricopharyngeal narrowing segment in radiographic contrast swallow examination. The patient was treated successfully by cricopharyngeal myotomy, achieving long-term relief in our 4 years of follow up.

**Conclusions:**

Our case of delayed cricopharyngal dysfunction after radical thyroidectomy and medialization thyroplasty shows that it is important to follow up swallowing functions after patients with UVCI undergo medialization thyroplasty. In the event of delayed manifestation of cricopharyngeal function, it can still be treated successfully by cricoharyngeal myotomy, achieving long term relief of dysphagia.

## Background

Unilateral vocal cord immobility (UVCI) caused by recurrent laryngeal nerve injury often results in problems with dysphonia, aspiration, and swallowing difficulties. It is often the phonatory problems that prompt patients to seek medical attention [1], and medialization thyroplasty is generally considered the phonosurgical procedure for voice augmentation if the cause of phonatory problem is irreversible. Aspiration is the second most common morbidity in patients with UVCI^1^, and swallowing dysfunction has also been noted to be associated with glottis insufficiency. Cricopharyngeal dysfunction is a disorder caused by failure of the cricopharyngeus muscle in upper esophageal sphincter (UES) to relax during swallowing and thereby causing oropharyngeal dysphagia [2]. Open cricopharyngeal myotomy, performed through a cervical approach, has been successful in relieving oropharyngeal dysfunction resulted dysphagia in a variety of structural, myogenic, neurogenic, and idiopathic disorders [2]. It also provides longer relieve on oropharyngeal dysfunction than botox injection which is common alternative treatment option.

We report a case who suffered from left unilateral vocal cord immobility after radical thyroidectomy with neck lymph nodes dissection and type I thyroplasty for the treatment of phonatory insufficiency. The patient developed dysphagia and aspiration 2 years after the surgeries, and the patient was treated successfully by cricopharyngeal myotomy, achieving long-term relief of symptoms in our 4 years of follow up. This case adds to our experience that oropharyngeal dysfunction can be a delayed manifestation of head and neck surgeries, and appropriate surgical intervention after onset of symptoms can still achieve ideal relieve without adverse effects.

## Case presentation

**Fig. 1:**
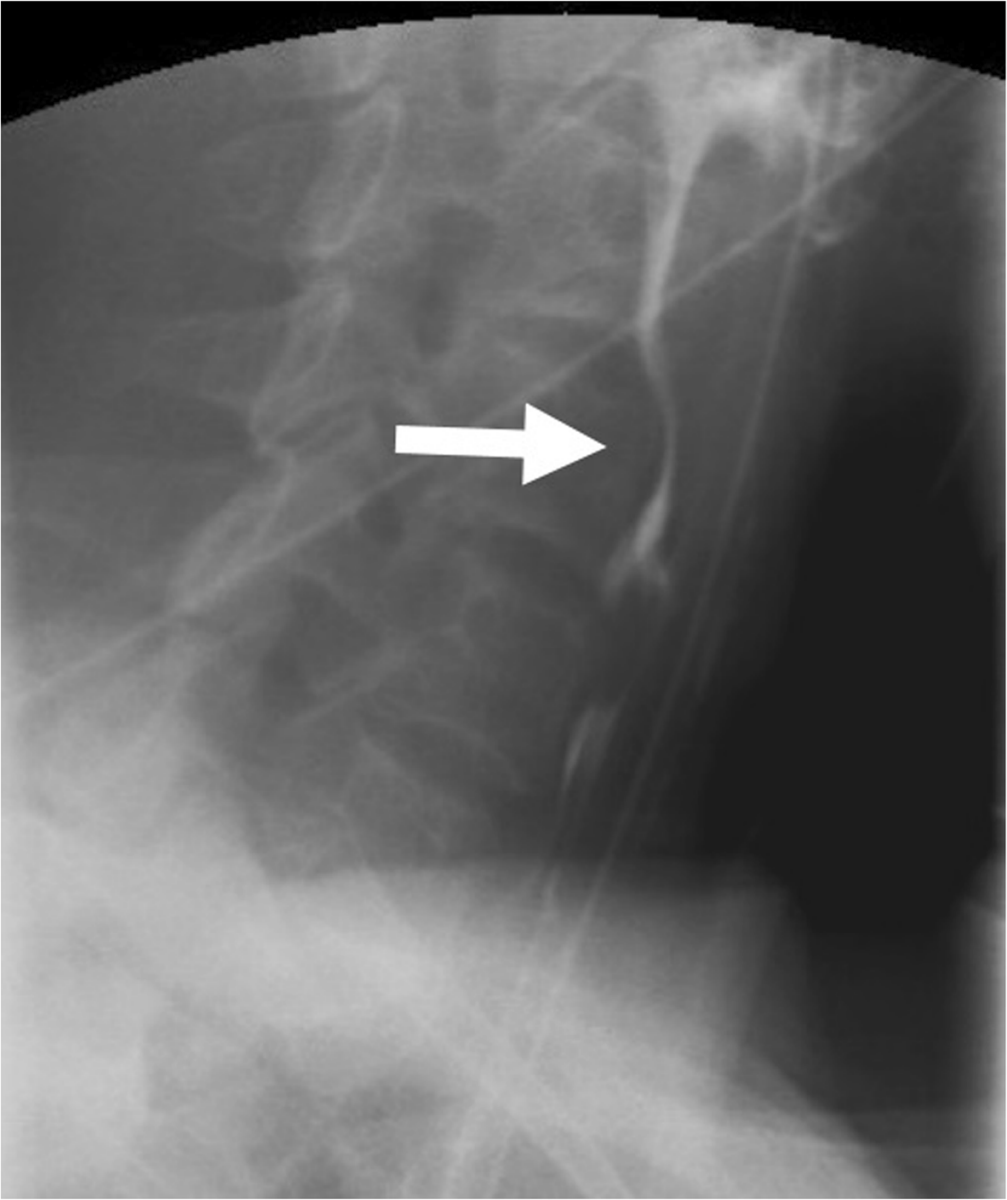
A Cricopharyngeal narrowing was seen in the lateral projected barium radiographic study*

A 62-year-old woman was diagnosed of thyroid cancer and received total thyroidectomy with left neck lymph nodes dissection (level II, III, IV, IV, and VI). The patient suffered left unilateral vocal cord immobility after the surgery, and she received type I thyroplasty with vocal cord medialization 1 month after the thyroid and neck dissection surgery as treatment for her phonatory insufficiency. The patient did not receive further chemotherapy or radiation therapy, and no evidence of residual thyroid cancer or recurrence had been noted in her follow ups. There was no complaint of swallowing difficulty and aspiration before and after the neck operations. However, it was 2 years after the last previous operation of thyroplasty that she progressively developed oropharyngeal dysphagia with frequent coughing and aspiration. Physical and neurological examinations were unremarkable. Endoscopy and cervical computed tomography (CT) revealed no structural abnormality. A videotaped barium radiographic study (videoesophagraphy) showed a narrowing segment at the level of the cricopharyngeus muscle with partial dilation of the proximal esophageal segment (Fig. 1).

*A bottle of liquid barium sulfate was diluted with 60 ml of water

**Fig. 2:**
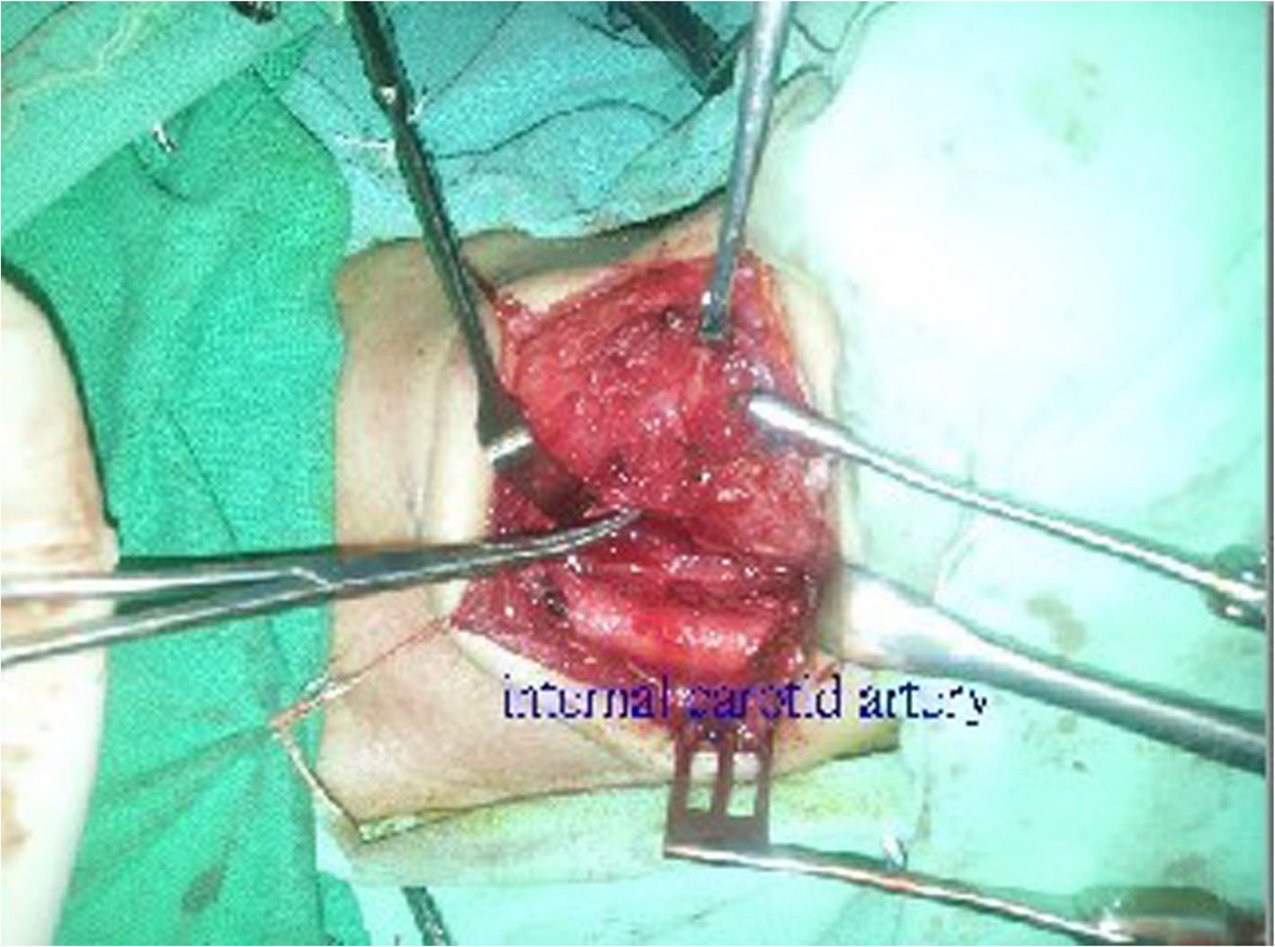
Cricopharyngeal muscle was transected and the cut ends were everted outward for suture fixation to prevent spontaneous re-anastomosis of the surgical edges

**Fig. 3:**
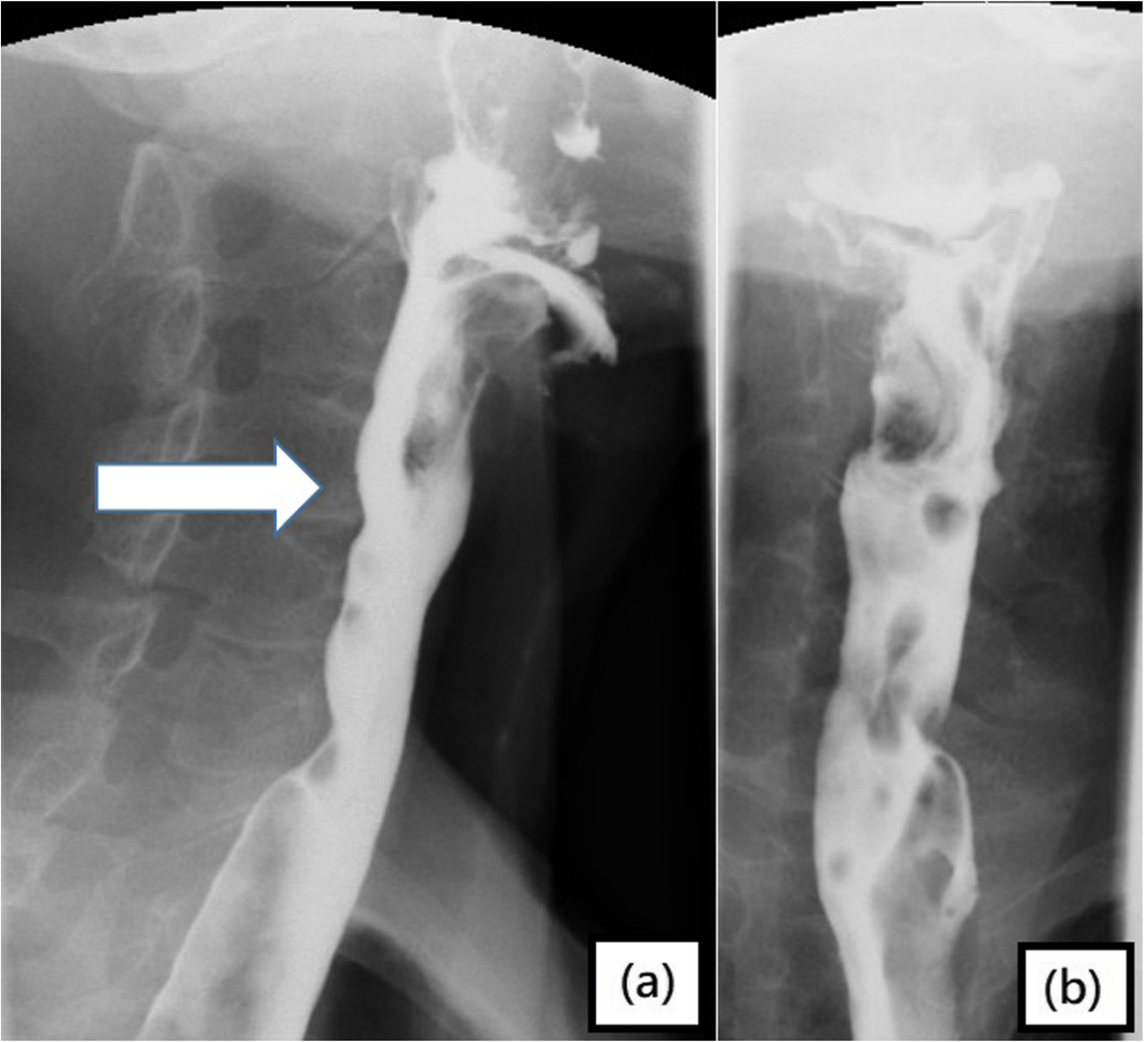
Dilatation of proximal cervical segment was noted on: **a** lateral and **b** anteroposterior radiographs of barium radiographic test* after cricopharyngeal myotomy

After cricopharyngeal dysfunction was confirmed by barium radiographic study, operation was arranged for the patient in which cricopharyngeal myotomy was done (Fig. 2). Immediately after the operation, the dysphagia resolved remarkably. Postoperative videoesophagraphy (Fig. 3) showed improved cricopharyngeal opening, and there was no sign of the cricopharyngeal narrowing. The patient has remained asymptomatic for 4 years of follow-up, and no adverse side effect of the myotomy has been complained by the patient.

*A bottle of liquid barium sulfate was diluted with 60 ml of water

## Discussion and conclusions

The process of deglutition involves the reflex coordination of oral, pharyngeal, and laryngeal musculature. Aside from obvious disruption to the anatomical musculatures due to head and neck surgeries, a large number of dysphagia cases related to head and neck surgeries arise from injury to the nerves by inadvertently stretching, ligating, or transecting the complex network of nerves [3]. The presence of inflammation, malignancy, or radiotherapy for head and neck cancer can also increase the risk of nerve injury [3].

The vagus nerve, which is considered to be the important nerve in the larynx that controls the laryngeal musculature and the reflex coordination, gives rise to the recurrent laryngeal nerve (inferior laryngeal nerve) and the external branch of the superior laryngeal nerve (EB-SLN). After branching from the vagus nerve and coursing back to the larynx, the recurrent laryngeal nerve divides into an external branch providing motor function to the four intrinsic laryngeal muscles, and an internal branch with only sensory activity for the glottis. It has been well characterized that injury to the recurrent laryngeal nerve results in ipsilateral vocal immobilization which leads to impaired phonation, aspiration, and even breathing difficulty. Another nerve in the larynx that also contributes to deglutition process is superior laryngeal nerve. The superior laryngeal nerve consists of two branches: the internal laryngeal nerve (sensory), which supplies sensory fibers to the supraglottis, and the external laryngeal nerve (motor), which innervates the cricothyroid muscle. It is well understood that the activation of SLN tilts the thyroid cartilage and tenses the vocal cord by modifying the distance between the cricoid and the thyroid cartilages. Vocal fold tension and thickness influence the frequency of the vibration [4]. Injury to the nerve results in changes both to the quality of the voice, and the production of high pitched sound. However, injury to the SLN is less recognized than the injury to the recurrent laryngeal nerve, and SLN injury is believed to be the most commonly underestimated in head and neck and thyroid surgeries with reported prevalence varies widely from 0 to 58% [4]. Animal studies have demonstrated that unilateral transection of the SLN can alter the sequence of activity in the pharyngeal muscles, and these findings are identified as modulations of the central pattern generator for pharyngeal swallowing [5]. Signs of dysphagia can be recognized in dogs and have been described in both experimental and clinical studies [5].

Medialization thyroplasty in patients with unilateral vocal cord immobilization (UVCI) allows better apposition of the two vocal cords during phonation, and it is generally considered a phonosurgical procedure for voice augmentation. Patients with UVCI are also likely to have swallowing difficulty especially if it is related to recurrent laryngeal nerve damage, because recurrent laryngeal nerve at least partially innervates the proximal cervical esophagus [6]. Study by Paul W. Flint etc. reported 61% of UVCI patients had swallowing difficulties with greater severity of symptoms in superior laryngeal nerve/recurrent laryngeal nerve injury group [7]. Some studies have suggested that swallowing difficulty in UVCI patients may improve with vocal fold medialization [6], however, a recent study by Rachael E. Kammer showed that patients with UVCI and swallowing dysfunction does not benefit from medialization injection [8]. It is important to know that medialization thryroplasty may lose its effectiveness as the thyroarytenoid muscle atrophies with continued denervation [9]. This may lead to deterioration in glottic functions, resulting in not only dysphonia but also progression of dysphagia and aspiration. Several studies have recommended cricopharyngeal myotomy to be performed along with medialization thryroplasty for UVCI patients presented with swallowing difficulty [7].

It is highly probable that our case has suffered left recurrent laryngeal nerve injury with evidence of left vocal cord immobility after the neck surgery for thyroid cancer, and the patient showed hoarseness and aspiration episodes in correlation with left vocal palsy. However, it is hard to obtain objective evidence of superior laryngeal nerve injury or its wellness from the patient, because intraoperative identification of SLN is not routinely done, and there is no standard SLN examination postoperatively. It has become a matter of our concern because, from this case, we have unanswered questions that whether injury to the superior laryngeal nerve combined with injury to the recurrent laryngeal nerve would affect a patient’s dysphagia severity, and its’ correlation with corresponding treatment plans. Studies show that Zenker’s diverticulectomy and diverticlopexy are associated with high risks of recurrent laryngeal nerve paralysis, and surgical combination with cricopharygeal myotomy shows lower recurrence rate [10]. Although nerve identification is not routinely done in Zenker’s diverticulum surgeries, it strongly suggests that nerve injury can affect post-surgical swallowing function. We acknowledge that utilization of laryngeal electromyography (EMG) would have helped us to confirm and analyze the patient’s cricopharyngeal dysfunction, and it is one of the areas that our institute is developing to better understand different cases of cricopharyngeal dysfunction.

Although radiographic appearance of cricopharyngeal bar, or cricopharyngeal narrowing in our patient, does not directly corroborate criciopharyngeal dysfunction, cricopharyngeal muscle is the primary muscle of the upper esophageal sphincter (UES) which needs to relax to allow the bolus to enter the esophagus. If the UES opening is limited in width or impaired in coordination, truncation of the bolus and residue can lead to aspiration when the airway reopens for breathing. Cricopharyngeal muscle receives dual innervations from the glossopharyngeal nerve (CN IX) via the pharyngeal plexus and the recurrent laryngeal nerve (CN X). We have known that recurrent laryngeal nerve has been injured in our patient, therefore, it strongly suggests that cricopharyngeal narrow seen in our patient correlates with our patient’s dysphagia after eliminating structuration abnormality in the esophagus and pharynx. Treatment options for cricopharyngeal dysfunction include injection of botulinumm toxin, dilatations, and surgical myotomy of the muscle [2]. We arranged cricopharyngeal myotomy for the patient because cricopharyngeal structureal abnormality was noted in barium swallow test, and we knew that the patient suffered permanent injury to recurrent laryngeal nerve.

Our case of delayed cricopharyngal dysfunction after radical thyroidectomy with neck lymph nodes dissection and medialization thyroplasty shows that it is important to follow up swallowing functions after patients with UVCI undergo medialization thyroplasty. The procedure might cause progression of glottic insufficiency in swallowing. In the event of delayed manifestation of cricopharyngeal function after medialization thyroplasty can still be treated successfully by cricoharyngeal myotomy, achieving long term relief of dysphagia.

## Data Availability

Not applicable

## Abbreviations

CN IX: cranial nerve IX (glossopharyngeal nerve)
CN X: cranial nerve X (recurrent laryngeal nerve)
EB-SLN: external branch of the superior laryngeal nerve
SLN: superior laryngeal nerve
UES: upper esophageal sphincter
UVCI: unilateral vocal cord immobilization
